# Association of atopic dermatitis with depressive symptoms and suicidal behaviors among adolescents in Korea: the 2013 Korean Youth Risk Behavior Survey

**DOI:** 10.1186/s12888-016-1160-7

**Published:** 2017-01-03

**Authors:** Seulki Lee, Aesun Shin

**Affiliations:** 1 Seoul National University College of Medicine, Seoul, Korea; 2 Department of Preventive Medicine, Seoul National University College of Medicine, 103 Daehangno, Chongno-gu, Seoul, 110-799 Korea

**Keywords:** Adolescent atopic dermatitis, Depressive symptoms, Suicide ideation, Suicide planning, Suicide attempts

## Abstract

**Background:**

Atopic dermatitis (AD) is a chronic skin disease which has been known to negatively influence the mental health of patients. However, only a few studies have explored the prevalence of psychiatric problems among AD patients, particularly among adolescents. In this study, we aimed to assess the association of AD with depressive symptoms and suicidal behaviors among adolescents by analyzing data from the 2013 Korean Youth Risk Behavior Survey, a nationwide web-based survey.

**Methods:**

Data from 72,435 adolescent middle and high school students in Korea were analyzed. Students self-reported AD diagnosed by a doctor and yes-or-no answers to questions about depressive symptoms and suicide ideation, suicide planning, and suicide attempts were analyzed. Relationships between AD and depressive symptoms or suicidal behaviors were tested by logistic regression models after controlling for potential confounding factors.

**Results:**

The proportion of adolescents who had AD was 6.8%. The proportion of adolescents reporting depressive feelings was 31.0%, suicide ideation was 16.3%, suicide planning was 5.8%, and suicide attempts was 4.2%. Compared to adolescents without AD, adolescents with AD were significantly more likely to experience depressive feelings (odds ratio [OR]: 1.27, 95% confidence interval [Cl]: 1.19-1.36), suicide ideation (OR: 1.34, 95% Cl: 1.24-1.45), suicide planning (OR: 1.46, 95% Cl: 1.32-1.65), and suicide attempts (OR: 1.51, 95% Cl: 1.33-1.72). In the multivariate model, the relationships between AD and suicide ideation (OR: 1.26, 95% Cl:1.16-1.36), suicide planning (OR: 1.28, 95% Cl:1.14-1.44), and suicide attempt (OR: 1.29, 95% Cl:1.13-1.49) were statistically significant.

**Conclusion:**

Adolescents who have AD are associated with a higher prevalence of depression symptoms and suicidal behaviors. Adolescent AD patients may need interventions from clinicians and caregivers that use a holistic approach to prevent psychological comorbidities, although further research is needed to clarify this relationship.

## Background

Atopic dermatitis (AD) is a chronic inflammatory skin condition characterized by pruritic, erythematous lesions, which is typically localized on flexural areas, the face, or the hands and which usually manifests in early childhood [1]. The worldwide prevalence of AD is 10%-20%, and it has significantly increased during the last three decades [2]. In Korea, its prevalence is estimated to be about 10% in the pediatric population less than 6 years of age and about 3% among adults [3]. In the Eighth Korean Youth Risk Behavior Survey (KYRBS), a nation-wide web-based survey, conducted by the Korea Centers for Disease Control and Prevention in 2012, 24% of adolescents responded that they had ever been diagnosed with AD [4]. The pathophysiological mechanisms of AD are not well understood; however cumulative evidence has shown that interactions between systemic and local immunologic responses, defects in skin barrier function, susceptibility genes, and the host’s environment are involved in producing AD symptoms [2]. The risk factors that contribute to an increased prevalence of AD include female sex [5], young age [5], higher socioeconomic status [6], smaller family size [7], and residence in an urban environment [8].

Dermatologic disorders such as AD are not fatal, but the resulting irritation, sleep disturbances due to itching and sensation of heat increase AD patients’ psychological burden [9]. Additionally, people often comment on their appearance, and many of these patients feel socially isolated, all of which may decrease their quality of life [10]. It has been reported that AD patients feel relatively high levels of anxiety and depressive symptoms [11], and chronic dermatologic illnesses can be associated with depression and suicide ideation [12]. In the late 1990s, a case-control study reported that children aged 5-15 years with moderate or severe AD scored twice as high as children without AD on a psychological disturbances scale. [9]. In another study, patients aged 16-56 years scored higher than normal controls on a self-reported depression scale, and their scores increased according to the severity of their AD [11]. Several cross-sectional, population-based studies of adult women and men have consistently reported an elevated risk for depression among participants with AD, and the strengths of the associations varied depending on the severity of AD symptoms (odds ratios [ORs]: 1.31-6.28) [13–15].

A large population-based survey in 2012 based on children aged under 18 years in the United States showed higher odds ratios (ORs) for depression in children with AD compared to children without AD (OR: 1.81, 95% Cl: 1.33-2.46) [16]. The same study also found a dose-response relationship between the prevalence of depression and the severity of AD symptoms. However, the study did not assess the associations between AD and suicidal behaviors.

The period of adolescence is known to be associated with high levels of psychological burden and vulnerability to depression [17–19]. Depression is one of the most significant risk factors for suicide. [20–22]. In 2012, it was reported that the prevalence of depressive symptoms experienced by the Korean population over 19 years of age was 12.9% [23], whereas the prevalence among adolescents was 30.5% [4]. In addition, the proportion of adolescents who experienced suicide ideation was 18.3%, suicide planning was 6.3%, and suicide attempts was 4.1% [4]. According to a report concerning suicide rate in Korea, the suicide rate in the children and youth category (10-24 years old) has been increased by 47% from 2000 to 2010, while the average suicide rate of Organisation for Economic Co-operation and Development (OECD) countries decreased by 16% during the same period [24].

Although there have been concerns about mental health among adolescents, very few studies have attempted to examine the relationship between AD and psychiatric symptoms in the general adolescent population aged 10-18 years. In a study using the Eighth KYRBS, the authors reported on the associations between atopic dermatitis and suicidal behaviors in girls. They concluded that the higher risk of suicidal behavior in girls was due to their higher prevalence of distorted weight perception. The association with depressive symptoms was not described, and the strength of the relationship was somewhat weak, which might be attributed to the question used to assess AD in the Eighth KYRBS (“*Have you ever been diagnosed with atopic dermatitis by a doctor at any point in your life?*”). The question asked whether the participants had ever been diagnosed with AD, regardless of their current symptoms, which led to the higher rate of AD mentioned above (24%) [25]. In the Ninth KYRBS, an additional question on whether the participants had been diagnosed in the past 1 year was added, which is more relevant for describing current mental health concerns and its association with AD. In this study, we assessed the association between AD and symptoms of depression or suicidal behaviors in an adolescent population living in Korea using data from the Ninth KYRBS.

## Methods

### Data source

This study analyzed data from Ninth KYRBS 2013 [26], which is conducted every year under Article 19 of the National Health Promotion Law by the Korean Ministry of Health and Welfare, Korean Ministry of Education at the Korea Centers for Disease Control and Prevention. The survey comprises 15 sections (Smoking, Drinking, Physical activity, Diet, Obesity and weight control, Mental health, Injuries and safety awareness, Oral health, Personal hygiene, Drug use, Sexual behavior, Atopy and asthma, Internet addiction, Socioeconomic status, and Violence) that were developed based on other studies conducted by the Korea Centers for Disease Control and Prevention. The Ninth KYRBS gathered responses from sample population, who were 75,149 middle and high school students in Korea, about risk behaviors such as cigarette smoking and alcohol consumption. The study has been granted an exemption from ethics approval, since it used open-access public data (exemption approval: Seoul National University Hospital IRB # 1508-110-696).

### Study population

The study population was defined as middle or high school students living in Korea in April 2012. The population was stratified by 43 regions and school type, and then the number of sample schools in each region was determined according to the composition of the target population. Using stratified cluster sampling, 800 sample schools were systematically selected. Then sample classes were selected randomly and every student in the class, except for exceptional cases such as long-term absentee, students with disability, were included in the final dataset. The survey targeted 75,149 students from 400 middle schools and 400 high schools, and the participation rate was 96.4%. The reasons for non-participation were not available. A total of 72,435 students at 799 schools were included in the analysis, and there was no non-response because the survey system was designed to answer every question. However, some of the data was considered as missing value if there was any logical error or if it was outlier. For example, the respondent from a boy school answered as ‘Female’, then the answer was processed as a missing value. The proportion of missing values was about 2%.

### Study variables

The diagnosis of AD was evaluated by an affirmative response to the question “*During the past 12 months, have you ever been told by a doctor or other health care provider that you had atopic dermatitis*?”. Depression symptoms, suicide ideation, suicide planning, and suicide attempts were assessed by the following questions: “*During the past 12 months*, *did you ever feel so sad or hopeless almost every day for two weeks or more in a row that you stopped doing some usual activities*?”, *“During the past 12 months, have you ever seriously thought of committing suicide?”*, *“During the past 12 months, have you ever made a plan about how you would attempt suicide?”*, and, *“During the past 12 months, have you ever attempted suicide?”*.

The potential confounders considered were sex, age (12 ~ 14 years (middle school)/15 ~ 17 years (high school))[2, 27], area of residence (rural/urban area)[8, 28], family affluence (low/middle/high), parental education (<high school graduation/high school graduation/>college graduation)[6, 29], sleep duration (<5 hours/5-7 hours/>7 hours), sleep satisfaction (well enough/enough/not enough) [9, 30], violence (never experienced/experienced ≥ 1 time) [31, 32], and alcohol consumption (individuals with problem drinking/drinker/non-drinker) [33, 34] which were known to be associated with AD and depression. Additionally, only a little evidence supports the association with AD, vigorous physical activity (do/do not ≥ 1 per week) [35], and smoking (non-smoker/smoke <10 cigarettes per day/smoke ≥10 cigarettes per day) [36] were also considered as the confounders.

All questions and their multiple-choice answers for each issue were set by the KYRBS 2013 [37]. We categorized the answers based on known knowledge about potential relationships with AD or depression. Respondents who lived in the county or rural areas were categorized as ‘Rural’; those who lived in small, middle-sized or large cities were categorized as ‘Urban’. Family affluence was calculated by a four-item measure of family wealth that considered the number of family cars, holidays, personal computers, and bedrooms; a score of 0-2 was categorized as ‘Low’, 3-5 was categorized as ‘Middle’, and over 6 was categorized as ‘High’ [38]. The respondents’ father’s and mother’s education were considered separately. Vigorous physical activity was defined as exercising for more than 20 minutes causing rapid breathing and a substantial increase in heart rate [26]. Because exercise duration rather than frequency is a critical factor for developing depressive feelings [39], participation in vigorous physical activity was categorized as ‘Do ≥ 1 per week’ or ‘Do not’. For smoking, heavy smokers were defined as those who smoked over 10 cigarettes per day on average during the last 30 days [26]. For alcohol consumption, ‘drinker’ was defined as respondents who drank at least 1 shot glass of alcohol during the last 30 days, and ‘non-drinker’ was defined as those who did not drink during the last 12 months or had no experience drinking alcohol [26]. Since it is well known that alcohol is correlated with depression [33], we further categorized respondents who drank alcohol and who experienced alcohol-related problems, such as dependence on alcohol, loss of memory or causing trouble with other people when drunk, during the last 12 months as an ‘individuals with problem drinking’. [26]. Sleep duration was calculated by using the bedtime and wake-up time reported by respondents during the last 7 days. There were 5 choices (well enough/enough/so-so/not enough/never enough) for the sleep satisfaction question [26]. The first two were grouped into a single category (‘enough’), as were the last two (‘not enough’). Respondents were categorized as having experienced violence if they reported experiencing physical and/or psychological violence such as bullying or threatening by others at least once during the last 12 months [26].

### Statistical methods

The univariate analysis was conducted to estimate the prevalence of AD and each mental health condition by the various sociodemographic factors. The association of AD with the presence of depression symptoms and suicidal behaviors was assessed by using the χ^2^ test between the AD and non-AD groups. Logistic regression models were used to further assess the relationship between the variables by calculating the ORs and their 95% confidence intervals (CIs). Of the potential confounding factors, those that were significantly associated with AD were entered one by one into the regression model, which included AD as an independent variable. The factors that substantially changed the ORs (over 10%) between AD and depression symptoms, suicidal ideation, or suicidal planning were planned to be included in the multivariate logistic regression model; however, none of the factors had a significant impact on the results. Therefore, only sex and age were entered in the logistic regression model. Additionally, the results of the multivariate logistic regression model that includes other potential confounders and depression symptom, with the exception of participation in physical activity and cigarette smoking, were presented. Because the associations between AD and mental health outcomes were insignificant among boys in a previous study, the ORs were further assessed stratified by sex to determine if there were any differences between both sexes. All analyses were conducted by applying strata, cluster, and weight procedures to properly estimate the variance and were performed with SAS version 9.2 (SAS Institute, Cary, North Carolina, United States).

## Results

The prevalence of AD diagnosed during the past year among the adolescent study population was 6.8%. The proportion of adolescents reporting having had depression symptoms was 31.0%, suicide ideation was 16.3%, suicide planning was 5.8%, and suicide attempts was 4.2%. The characteristics of the study population are shown in Table 1. Those with AD had a higher prevalence of having female gender (*P* < 0.001), living in urban area (*P* = 0.002), higher family affluence score (*P* < 0.001), parents with higher educational attainment (*P* < 0.001 for father’s education; *P* = 0.009 for mother’s education), drinking problem, (*P* = 0.002), shorter sleeping duration (*P* < 0.001), unsatisfactory sleep (*P* < 0.001), and violence experience (*P* < 0.001). There were no significant differences between middle and high school students. There were no significant differences between middle and high school students.

**Table 1:** Sociodemographic characteristics of 72,435 adolescents with and without atopic dermatitis, the Ninth Korean Youth Risk Behavior Survey (2013), N (%)

Variables	Adolescents without atopic dermatitis	Adolescents with atopic dermatitis	*P*-value
Sex			
Male	34,555 (52.9)	2,100 (44.5)	<0.001
Female	32,976 (47.1)	2,804 (55.5)	
Age			
12–14 years (Middle school)	34,021 (48.7)	2,509 (49.7)	0.101
15–17 years (High school)	33,510 (51.3)	2,395 (50.3)	
Residence^a^			
Rural area	8,328 (6.9)	530 (6.2)	0.002
Urban area	59,203 (93.1)	4,374 (93.8)	
Family affluence^b^			
Low	8,021 (11.4)	529 (10.4)	<0.001
Middle	35,285 (52.8)	2,482 (50.0)	
High	24,234 (36.8)	1,893 (39.6)	
Father’s educational attainment			
<High school graduation	2,581 (3.8)	170 (3.5)	0.009
High school graduation	22,283 (33.0)	1,652 (33.7)	
> College graduation	29,173 (43.2)	2,189 (44.6)	
Missing^e^	13,494 (20.0)	893 (18.2)	
Mother’s educational attainment			
< High school graduation	2,396 (3.6)	174 (3.6)	<0.001
High school graduation	28,020 (41.2)	2,110 (43.0)	
> College graduation	23,981 (35.5)	1,794 (36.6)	
Missing^e^	13,134 (19.5)	826 (16.8)	
Participation in physical activity^c^			
Do not	16,050 (23.8)	1,143 (23.2)	0.465
Do ≥ 1 per week	51,481 (76.2)	3,761 (76.8)	
Cigarette smoking			
Non-smoker	60,934 (90.3)	4,407 (89.6)	0.151
<10 /day	5,296 (7.8)	383 (8.0)	
≥10 /day	1,301 (1.9)	114 (2.4)	
Alcohol consumption			
Non-drinker	56,425 (83.8)	4,044 (83.0)	0.002
Drinker	6,552 (9.7)	464 (9.3)	
Individuals with problem drinking^d^	4,554 (6.5)	396 (7.7)	
Sleep duration			
<5 h	14,557 (21.2)	1,162 (23.4)	<0.001
5~7 h	24,350 (37.1)	1,763 (37.0)	
7 h<	22,013 (31.5)	1,471 (28.9)	
Missing^e^	6,611 (10.2)	508 (10.7)	
Sleep satisfaction			
Well enough	17,356 (25.6)	1,189 (24.2)	<0.001
Enough	21,791 (32.4)	1,442 (29.5)	
Not enough	28,384 (42.0)	2,273 (46.3)	
Violence			
Never experienced	65,307 (96.7)	4,688 (95.8)	<0.001
Experienced ≥ 1 time	2,224 (3.3)	216 (4.2)	

^a^ Urban area: small, medium, or big city

^b^ Family Affluence Score = number of cars + holidays + personal computers + bedrooms. 0–2: Low, 3–5: Middle, >6: High

^c^ An activity such as jogging, playing sports over 20 min causing rapid breathing and a substantial increase in heart rate

^d^ Those who experienced problem drinking during the last 12 months

^e^ Those who did not answer

In the same way, the distribution of sociodemographic characteristics among those with and without each mental health condition was assessed as shown in Table 2. All differences were significant except for those of suicidal behaviors between residence area and physical activity. Those who had depression symptoms and suicidal behaviors had a higher prevalence of being female gender, older age, lower family affluence score, having parents with lower educational attainment, smoking problem, drinking problem, short sleeping period, unsatisfactory sleep, and violence experience (*P* < 0.01 for all factors).

**Table 2:** Sociodemographic characteristics of 72,435 adolescents with and without each mental health condition, the Ninth Korean Youth Risk Behavior Survey (2013), N (%)^a^ Urban area: small, medium, or big city

Variables	Had depression symptoms		*P*-value	Suicide ideation		*P*-value	Suicide planning		*P*-value	Suicide attempt		*P*-value
	No	Yes		No	Yes		No	Yes		No	Yes	
Sex												
Male	27462 (56.6)	9193 (42.7)	<0.001	31885 (54.5)	4770 (41.3)	<0.001	34900 (52.8)	1755 (43.6)	<0.001	35590 (53.0)	1065 (35.8)	<0.001
Female	22543 (43.4)	13237 (57.3)		28480 (45.5)	7300 (58.7)		33367 (47.2)	2413 (56.4)		33824 (47.0)	1956 (64.2)	
Age												
12–14 years (Middle school)	26012 (50.1)	10518 (45.8)	<0.001	30186 (48.2)	6344 (51.7)	<0.001	34099 (48.2)	2431 (57.6)	<0.001	34680 (48.2)	1850 (61.1)	<0.001
15–17 years (High school)	23993 (50.0)	11912 (54.2)		30179 (51.8)	5726 (48.3)		34168 (51.8)	1737 (42.4)		34734 (51.8)	1171 (38.9)	
Residence^a^												
Rural area	6087 (6.8)	2771 (7.1)	<0.001	7428 (6.9)	1430 (6.8)	0.796	8327 (6.9)	531 (7.4)	0.307	8500 (6.9)	358 (6.9)	0.990
Urban area	43918 (93.2)	19659 (92.9)		52937 (93.1)	10640 (93.2)		59940 (93.1)	3637 (92.6)		60914 (93.1)	2663 (93.1)	
Family affluence^b^												
Low	5720 (11.0)	2821 (12.1)	<0.001	6983 (11.1)	1558 (12.2)	0.007	7975 (11.2)	566 (13.0)	<0.001	8117 (11.2)	424 (13.7)	<0.001
Middle	26116 (51.7)	11651 (51.6)		31594 (51.8)	6173 (50.7)		35719 (51.8)	2048 (49.0)		36328 (51.9)	1439 (46.9)	
High	18169 (37.3)	7958 (36.3)		21788 (37.0)	4339 (37.1)		24573 (37.0)	1554 (37.9)		24969 (36.9)	1158 (39.4)	
Father’s educational attainment												
<High school graduation	1814 (3.2)	9614 (45.1)	<0.001	2231 (3.3)	520 (3.8)	0.0018	2558 (3.3)	193 (4.1)	<0.001	2621 (3.3)	130 (3.9)	<0.001
High school graduation	16385 (31.6)	7550 (32.5)		20063 (32.1)	3872 (31.0)		22720 (32.1)	1215 (28.2)		23023 (32.0)	912 (29.1)	
>College graduation	21748 (46.1)	937 (3.7)		26172 (45.9)	5190 (45.4)		29603 (45.9)	1759 (44.4)		30155 (46.0)	1207 (42.6)	
Missing^e^	10058 (19.1)	4329 (18.6)		11899 (18.8)	2488 (19.7)		13386 (18.7)	1001 (23.3)		13615 (18.7)	772 (24.5)	
Mother’s educational attainment												
<High school graduation	1682 (3.0)	7865 (36.8)	<0.001	2063 (3.0)	507 (3.9)	<0.001	2400 (3.2)	170 (3.9)	<0.001	2459 (3.2)	111 (3.6)	<0.001
High school graduation	20612 (41.1)	9518 (41.9)		25238 (41.6)	4892 (40.0)		28573 (41.6)	1557 (37.1)		29013 (41.5)	1117 (36.7)	
>College graduation	17910 (37.7)	888 (3.7)		21515 (37.4)	4260 (37.3)		24304 (37.4)	1471 (37.1)		24765 (37.5)	1010 (35.4)	
Missing^e^	9801 (18.3)	4159 (17.7)		11549 (18.0)	2411 (18.8)		12990 (17.9)	970 (21.8)		13177 (17.9)	783 (24.2)	
Participation in physical activity^c^												
Do not	11719 (23.5)	5474 (24.5)	<0.001	14213 (23.7)	2980 (24.3)	0.165	16244 (23.8)	949 (22.6)	0.1554	16511 (23.8)	682 (22.7)	0.224
Do ≥ 1 per week	38286 (76.5)	16956 (75.5)		46152 (76.3)	9090 (75.7)		52023 (76.2)	3219 (77.4)		52903 (76.2)	2339 (77.3)	
Cigarette smoking												
Non-smoker	46152 (92.3)	19189 (85.7)	<0.001	55207 (91.4)	10134 (84.4)	<0.001	62009 (90.8)	3332 (80.4)	<0.001	63004 (90.8)	2337 (78.2)	<0.001
<10 /day	3176 (6.4)	2503 (11.1)		4211 (7.0)	1468 (11.9)		5095 (7.5)	584 (13.5)		5218 (7.5)	461 (14.6)	
≥10 /day	677 (1.3)	738 (3.2)		947 (1.6)	468 (3.7)		1163 (1.7)	252 (6.2)		1192 (1.7)	223 (7.3)	
Alcohol consumption												
Non-drinker	43211 (86.6)	17258 (77.4)	<0.001	51392 (85.3)	9077 (75.7)	<0.001	57484 (84.5)	2985 (72.2)	<0.001	58376 (84.3)	2093 (69.9)	<0.001
Drinker	4483 (9.0)	2533 (11.3)		5583 (9.2)	1433 (12.1)		6512 (9.5)	504 (12.3)		6652 (9.6)	364 (12.3)	
Individuals with problem drinking^d^	2311 (4.4)	2639 (11.3)		3390 (5.5)	1560 (12.2)		4271 (6.0)	679 (15.5)		4386 (6.1)	564 (17.8)	
Sleep duration												
<5 h	10124 (20.0)	5595 (24.6)	<0.001	12481 (20.3)	3238 (26.8)	<0.001	14415 (20.8)	1304 (31.5)	<0.001	14725 (20.9)	994 (33.4)	<0.001
5~7 h	17719 (36.7)	8394 (38.0)		21807 (37.3)	4306 (35.9)		24822 (37.5)	1291 (31.1)		25191 (37.4)	922 (30.5)	
7 h<	17770 (34.2)	5714 (24.8)		20376 (32.5)	3108 (25.3)		22367 (31.6)	1117 (26.2)		22702 (31.5)	782 (25.3)	
Missing^e^	4392 (9.2)	2727 (12.6)		5701 (9.9)	1418 (12.1)		6663 (10.2)	456 (11.2)		6796 (10.2)	323 (10.8)	
Sleep satisfaction												
Well enough	14818 (29.4)	3727 (16.8)	<0.001	16662 (27.5)	1883 (15.9)	<0.001	17817 (26.0)	728 (17.7)	<0.001	18050 (25.9)	495 (17.2)	<0.001
Enough	16788 (33.6)	6445 (28.9)		20035 (33.3)	3198 (26.6)		22202 (32.6)	1031 (25.2)		22487 (32.5)	746 (24.4)	
Not enough	18399 (37.0)	12258 (54.3)		23668 (39.3)	6989 (57.6)		28248 (41.4)	2409 (57.1)		28877 (41.6)	1780 (58.4)	
Violence												
Never experienced	48927 (97.9)	21068 (94.0)	<0.001	58900 (97.6)	11095 (92.1)	<0.001	66417 (97.3)	3578 (85.7)	<0.001	67456 (97.2)	2539 (83.9)	<0.001
Experienced ≥1 time	1078 (2.1)	1362 (6.0)		1465 (2.4)	975 (7.9)		1850 (2.7)	590 (14.3)		1958 (2.8)	482 (16.1)	

^a^ Urban area: small, medium, or big city

^b^ Family Affluence Score = number of cars + holidays + personal computers + bedrooms. 0–2: Low, 3–5: Middle, >6: High

^c^ An activity such as jogging, playing sports over 20 min causing rapid breathing and a substantial increase in heart rate

^d^ Those who experienced problem drinking during the last 12 months

^e^ Those who did not answer

Associations between AD and ‘had depression symptoms’ are shown in Table 3. Among adolescents with AD, there were higher prevalences of those who had felt sadness and hopelessness (OR: 1.28, 95% CI: 1.20–1.37), considered suicide (OR: 1.31, 95% CI: 1.21-1.42), planned suicide (OR: 1.42, 95% CI: 1.26-1.61), or attempted suicide (OR: 1.51, 95% CI: 1.32-1.73) than adolescents without AD. When other potential confounders were included in the final logistic regression model, the adjustment had little impact on the association of AD with having depression symptoms (OR: 1.24, 95% Cl: 1.15-1.34). On the other hand, the confounders significantly affected the relationship between AD and suicide ideation (OR: 1.23, 95% Cl:1.13-1.35), suicide planning (OR: 1.29, 95% Cl:1.13-1.48), and suicide attempt (OR: 1.31, 95% Cl:1.12-1.52).

**Table 3:** Associations between atopic dermatitis (AD) with mental health variables among 72,435 Korean adolescents

	Had depression symptoms^a^		OR^b^ (95% Cl)	Suicide ideation		OR^b^ (95% Cl)	Suicide planning		OR^b^ (95% Cl)	Suicide attempt		OR^b^ (95% Cl)
	No, N	Yes, N		No, N	Yes, N		No, N	Yes, N		No, N	Yes, N	
Total												
AD												
No	46,914	20,617	1.0	56,505	11,026	1.0	63,753	3,778	1.0	64,810	2,721	1.0
Yes	3,091	1,813	1.24 (1.15-1.34)	3,860	1,044	1.23 (1.13-1.35)	4,514	390	1.29 (1.13-1.48)	4,604	300	1.31 (1.12-1.52)
Male												
AD												
No	25,985	8,570	1.0	30,127	4,428	1.0	32,939	1,616	1.0	33,579	976	1.0
Yes	1,477	623	1.21 (1.07-1.37)	1,758	342	1.20 (1.02-1.40)	1,961	139	1.23 (0.99-1.52)	2,011	89	1.37 (1.04-1.80)
Female												
AD												
No	20,929	12,047	1.0	26,378	6,598	1.0	30,814	2,162	1.0	31,231	1,745	1.0
Yes	1,614	1,190	1.25 (1.14-1.38)	2,102	702	1.25 (1.12-1.39)	2,553	251	1.33 (1.13-1.57)	2,593	211	1.28 (1.08-1.53)

^a^Among various symptoms of depression, this includes feelings of sadness or hopelessness hampering daily activities for over 2 weeks

^b^Odds ratio adjusted for sex, age, residence, family affluence, father’s education attainment, mother’s education attainment, alcohol consumption, sleep duration, sleep satisfaction, violence

The relationship was analyzed by stratifying data by sex (Table 4), and similar OR values were observed with regard to depressive symptoms (OR: 1.21, 95% Cl: 1.07-1.37 for male, OR: 1.25, 95% Cl: 1.14-1.38 for female), suicide ideation (OR: 1.20, 95% Cl: 1.02-1.40 for male, OR: 1.25, 95% Cl: 1.12-1.39 for female), suicide planning (OR: 1.23, 95% Cl:0.99-1.52 for male, OR: 1.33, 95% Cl; 1.13-1.57 for female), and suicide attempt (OR: 1.37, 95% Cl: 1.04-1.80 for male, OR: 1.28, 95% Cl: 1.08-1.53 for female). In addition, interaction p-values for sex on the associations between AD and mental health variables were not statistically significant (data not shown).

**Table 4:** Associations between atopic dermatitis with depressive symptoms and suicidal behaviors among 72,435 Korean adolescents, stratified by sex

		Male			Female		
		Diagnosed atopic dermatitis during the past 12 months		OR^b^ (95% Cl)	Diagnosed atopic dermatitis during the past 12 months		OR^b^ (95% Cl)
		No, N (%)	Yes, N (%)		No, N (%)	Yes, N (%)	
Had depression symptoms^a^	No	25,985 (75.1)	1,477 (70.5)	1.0 (ref) 1.21 (1.07–1.37)	20,929 (63.4)	1,614 (57.4)	1.0 (ref) 1.25 (1.14–1.38)
	Yes	8,570 (24.9)	623 (29.5)		12,047 (36.6)	1,190 (42.6)	
Suicide ideation	No	30,127 (87.1)	1,758 (84.1)	1.0 (ref) 1.20 (1.02–1.40)	26,378 (80.0)	2,102 (75.0)	1.0 (ref) 1.25 (1.12–1.39)
	Yes	4,428 (12.9)	342 (15.9)		6,598 (20.0)	702 (25.0)	
Suicide planning	No	32,939 (95.4)	1,961 (93.8)	1.0 (ref) 1.23 (0.99–1.52)	30,814 (93.5)	2,553 (90.8)	1.0 (ref) 1.33 (1.13–1.57)
	Yes	1,616 (4.6)	139 (6.2)		2,162 (6.5)	251 (9.2)	
Suicide attempt	No	33,579 (97.3)	2,011 (95.8)	1.0 (ref) 1.37 (1.04–1.80)	31,231 (94.7)	2,593 (92.3)	1.0 (ref) 1.28 (1.08–1.53)
	Yes	976 (2.7)	89 (4.2)		1,745 (5.3)	211 (7.7)	

^a^ Among various symptoms of depression, this includes feelings of sadness or hopelessness hampering daily activities for over 2 weeks

^b^ Odds ratio adjusted for age, residence, family affluence, father’s education attainment, mother’s education attainment, alcohol consumption, sleep duration, sleep satisfaction, violence

When depression symptoms were included as a variable in the model of AD and suicidal behaviors, the associations became weaker but still remained significant (OR: 1.14, 95% Cl: 1.03-1.26 for suicide ideation, OR: 1.20, 95% Cl: 1.05-1.38 for suicide planning, OR: 1.22, 95% Cl: 1.04-1.42 for suicide attempt).

## Discussion

This study found an association between AD and depression symptoms and suicidal behaviors. The prevalence of those who ‘had depression symptoms’, have considered suicide, planned suicide and attempted suicide were higher among students with AD. And a similar strength of association was observed in both male and female students. These results confirm and expand upon previous findings on the relationship between mental health and AD.

The prevalence of AD in this study was 6.8% which is similar to the previous findings [3]. However, we found no significant differences in the prevalence of AD of between middle school students and high school students, which is not consistent with other findings that the prevalence of AD decreases with age [40–43]. This finding may be partly explained by the fact that students were living in a similar social environment and that the types of school did not exactly reflect the participants’ age.

Our findings on associations between AD and ‘had depression symptoms’ and suicidal thoughts are consistent with the results of previous population-based surveys [13–16]. However, in our study, the strength of the relationship was somewhat weaker than in other studies. This may be due to differences in the composition of the study population. For example, in a population-based study in the United States of a young population (<17 years) [16], 71% of the study population and about 41% of AD patients were under the age of 13. Consistent with the natural course of AD, patients tended to be younger and incidence decreased with age [42, 43]. Most other studies [9, 11, 12] were case-control studies with small-sized study population. In addition, unlike other studies, we used self-reported ‘had depression symptoms’, not depression diagnosed by a physician. These differences in research methods may explain the weaker relationship that we found.

Despite this, our results were statistically significant. Not every depressive patient develops suicidal behaviors, although 77% of students in our study who answered ‘yes’ to having depression symptoms also answered ‘yes’ to the question on suicide ideation. The ORs for each relationship increased as the clinical severity of the dependent variable increased (e.g., a suicide attempt was considered more severe than suicide ideation). One of the most significant contributing factors associated with adolescent suicide is major depression [20, 21]. Thus, interpreting these variables as interrelated depression symptoms can explain the trends found in this study, which needs further exploration. However, after adjusting for all potential confounding factors, the ORs for suicide ideation, planning, and attempt became weaker when compared to the sex and age-adjusted ORs. Additionally, the ORs for suicidal behaviors changed more substantially than the OR for ‘had depression symptoms’, which could mean that those confounding factors influenced the relationship when combined.

On the other hand, our results do not imply the direction of relationship. Although the mechanisms supporting the relationship between AD and depression are not well established, there are hypotheses supporting both directions. First, it is known that certain personality types and psychological stresses are contributing factors for developing AD. Stress-induced hypothalamic-pituitary-adrenal axis and autonomic nervous system responses followed by immunologic disturbances are the underlying mechanisms [44]. Thus, stressful conditions may increase the severity of AD symptoms. In addition, an individual’s psychological state may also affect AD, explaining the association of AD with depression symptoms and suicidal behaviors.

A study based on Finnish twins showed that AD patients are genetically vulnerable to depressive disorders [45]. This means that depression symptoms may not be a consequence of AD and that it may be difficult to prevent depression symptoms among AD patients. On the other hand, sleep loss caused by itching and scratching during the night may negatively affect patients’ quality of life and lead to the development of depression [46]^.^ Physical fatigue due to sleep disturbance during the night, as well as other factors including greater feelings of helplessness because of the illness and less social acceptance, have been found to be significantly related to high levels of psychological distress among AD patients [47]. This presents the possibility of developing treatment strategies for coping with the psychological distress of AD patients.

There are some fundamental limitations of this study, which should be considered when interpreting its results. First, we did not use AD as diagnosed by a caregiver or clinician, which may have led to a misclassification of AD. Thus, there is a possibility that the prevalence of AD in our study population was underestimated. Using a clinical diagnosis of AD would strengthen our findings. Meanwhile, self-reported allergies have been reported to have good correlations with medical records [48], and many studies have used the self-reported AD as a study variable. It is possible that the strength of the relationship between AD and experiencing depression symptoms was exaggerated. Due to the presence of depression symptoms, these individuals may have visited a doctor more often, which could have led to their AD being diagnosed more readily than AD patients without depression symptoms.

Second, we used self-reports of ‘had depression symptoms’ rather than a clinical diagnosis of depression. A two-week period of persistent depressive feelings or suicidal behaviors is considered to be a criterion but is not sufficient for a definite diagnosis of depression [49]. For example, the Diagnostic and Statistical Manual of Mental Disorders, 4th Edition (DSM-IV), includes questions on whether patients have had anhedonia, sleep disturbances, appetite changes, weight changes, fatigue, or a sense of worthlessness or inappropriate guilt [49]. Other studies have used a diagnosis of depression by a physician or a self-reported scale of depression symptoms based on questionnaires with multiple diagnostic questions. By using simplified diagnostic criteria, the prevalence of depression symptoms in our population may have been overestimated. The prevalence of diagnosed depression is generally lower than that of depression symptoms. For example, in Korea, the prevalence of depression is reported to be 3.6%-5.5% [50] compared with the 31% prevalence of depression symptoms in our study population [26]. However, there is also a possibility of underestimation. Depression in Asian countries tends to be expressed as somatized symptoms, rather than affective complaints [51]. Additionally, adolescent depression may present as substance abuse or disruptive behaviors, which we could not assess in this study, and it may be underdiagnosed due to a lack of healthcare [52]. As a result, the population we extracted as having depression symptoms and showing suicidal behaviors may not represent the population with depression disorders, and this could have over- or under-estimated the strength of the relationship between AD and depression.

Despite these limitations, this study was based on a large sample size from a nationwide general adolescent population survey that has shown good representativeness and generalizability. In addition, we used depressive feelings, suicide ideation, suicide planning, and suicide attempts as variables to indicate psychological distress and identify the consistency of relationships with AD. This finding is noteworthy, because it implies that AD may lead to destructive outcomes, such as major depressive disorders or suicide, which could be prevented, if suspected early. Depression is regarded as the most important risk factor for suicide [20–22], which is the fourth leading cause of death in Korea [53]. Levels of psychological distress are known to be relatively higher among adolescents, because the brain during this period undergoes significant development, which makes it exceptionally vulnerable to stress [19]. Adding to that, the prevalence of depressive symptoms and suicidal behaviors is higher among the Korean adolescent population compared with Western countries, which is thought to be the result of higher levels of pressure on studying and less time devoted to leisure [54, 55]. Evaluating and managing psychological problems needs to be emphasized more firmly in this kind of social environment.

## Conclusions

In conclusion, students with AD are prone to depressive feelings, and ideation, planning, attempts at suicide. Future longitudinal studies are needed to explore the direction of the relationship of interest. However, regardless of how AD is related to depression, depressive symptoms should be considered an important comorbidity of AD and taken into consideration in clinical practice. Clinicians should be cautious with regard to psychological issues when dealing with adolescent AD patients. There are no standardized clinical guidelines for treatment of psychological issues. However, patients with AD may benefit from multidisciplinary treatment plans that manage psychological symptoms. Treatment options that focus on fatigue reduction, changing patients’ pessimistic attitudes about their disease, and promoting social support have been developed and tested in a few studies and have found to be effective enough to relieve symptoms and improve the quality of life of children and adolescent patients with AD [47, 56, 57].

## Abbreviations

AD: Atopic dermatitis
KYRBS: the Korean Youth Risk Behavior Survey
